# Genetic Diversity and Population Structure of the Major Peanut (*Arachis hypogaea* L.) Cultivars Grown in China by SSR Markers

**DOI:** 10.1371/journal.pone.0088091

**Published:** 2014-02-10

**Authors:** Xiaoping Ren, Huifang Jiang, Zhongyuan Yan, Yuning Chen, Xiaojing Zhou, Li Huang, Yong Lei, Jiaquan Huang, Liying Yan, Yue Qi, Wenhui Wei, Boshou Liao

**Affiliations:** 1 Key Laboratory of the Biology and Genetic Improvement of Oil Crops, Ministry of Agriculture/Oil Crop Research Institute of the Chinese Academy of Agricultural Sciences, Wuhan, Hubei, China; 2 National Facility for Training Personnel in Life Sciences and Biotechnology, College of Life Sciences, Wuhan University, Wuhan, Hubei, China; New Mexico State University, United States of America

## Abstract

One hundred and forty-six highly polymorphic simple sequence repeat (SSR) markers were used to assess the genetic diversity and population structure of 196 peanut (*Arachis Hypogaea* L.) cultivars which had been extensively planted in different regions in China. These SSR markers amplified 440 polymorphic bands with an average of 2.99, and the average gene diversity index was 0.11. Eighty-six rare alleles with a frequency of less than 1% were identified in these cultivars. The largest *Fst* or genetic distance was found between the cultivars that adapted to the south regions and those to the north regions in China. A neighbor-joining tree of cultivars adapted to different ecological regions was constructed based on pairwise Nei’s genetic distances, which showed a significant difference between cultivars from the south and the north regions. A model-based population structure analysis divided these peanut cultivars into five subpopulations (P1a, P1b, P2, P3a and P3b). P1a and P1b included most the cultivars from the southern provinces including Guangdong, Guangxi and Fujian. P2 population consisted of the cultivars from Hubei province and parts from Shandong and Henan. P3a and P3b had cultivars from the northern provinces including Shandong, Anhui, Henan, Hebei, Jiangsu and the Yangtze River region including Sichuan province. The cluster analysis, PCoA and PCA based on the marker genotypes, revealed five distinct clusters for the entire population that were related to their germplasm regions. The results indicated that there were obvious genetic variations between cultivars from the south and the north, and there were distinct genetic differentiation among individual cultivars from the south and the north. Taken together, these results provided a molecular basis for understanding genetic diversity of Chinese peanut cultivars.

## Introduction

The cultivated peanut, *Arachis hypogaea* L., is an important oilseed and cash crop worldwide. It is one of the primary sources of vegetable oil and protein in developing countries. The peanut annual planting area is around 24 million hectares, with an annual production of nearly 35 million tons (http://apps.fao.org/cgi-bin/nph-db.pl?subset=agriculture). China is the largest peanut producer in the world with over 20% of planting area and more than 40% of production. During the last five decades, cultivated peanut had been subjected to intensive artificial selection, resulting in favorable changes in yield, disease resistance, biochemical composition and other agronomic traits [1]–[2]. Remarked progress had been made in peanut genetic improvement of yield [1]. However, more than 70% of the cultivars were affirmed to contain co-ancestors ‘Fuhuasheng’ and ‘Shitouqi’ by pedigree analysis [1]–[2], showing that the Chinese peanut cultivars could have a narrow genetic basis for peanut varieties.

The previous studies paid more attention on morphological and agronomic trait variations [3]–[8] and traits identification, e.g. diseases resistance [9]–[10], stress resistance [11]–[13] and high oleic acid content [14]. Very limited studies had been performed on the genetic diversity [14]–[16]. The knowledge of genetic diversity was critical for germplasm utilization in peanut breeding. To broaden the genetic variation of cultivated peanut in future breeding program, it is necessary to perform a more comprehensive of genetic diversity and population structure of the released varieties.

Compared with a large quantity of morphological and agronomic variation in peanut cultivars [3]–[8], however, extremely low levels of polymorphism in molecular were observed using restriction fragment length polymorphism (RFLP), randomly amplified polymorphic DNA (RAPD) and amplified fragment length polymorphisms (AFLP) [17]–[19]. Simple sequence repeats (SSRs), a kind of allele-specific and co-dominant molecular marker, had great potential in detecting the genetic diversity and relationships of organisms for their significant levels of allelic polymorphism [20]–[21]. With the rapid development of genomic-SSR and EST-SSR sequences [22]–[27], SSR marker had been widely applied in cultivar identity verification, diversity studies [21], [28]–[31], linkage map construction [32]–[37] and QTL analysis [38]–[39] in peanut.

Traditional cluster analysis could provide an easy and effective way in determining the genetic diversity of germplasm collections [40], and several other statistical systems including population structure [41], principal component analysis (PCA) [42] and principal coordinate analysis (PCoA) [43], they had been developed for analyzing the structure of natural populations through molecular markers, and widely used in rice [44]–[45], apple [46], sweet sorghum [47], wheat [48] and cucumber [49]. Cultivar populations were not only the natural populations but also the germplasm resources with specific desirable traits for genetic improvement. To analyze the genetic structure and to find out the excellent allelic variation, the cultivars with excellent allele had been an important system for the molecular design breeding program [50]. Estimating population structure is a necessary first step in association analysis, and is important to avoid false positives or spurious associations and to constrain association studies in natural populations [40]–[41], [51]. Analysis of 190 soybean accessions in China showed that the cultivar populations were composed of seven subpopulations, and linkage disequilibrium (LD) was detected extensively with syntenic and nonsyntenic markers [52]. A total of 29 SSR markers were used to analyze the genetic diversity of 150 accessions of cultivated rice from Korea, China, and Japan. The model-based structure analysis revealed the presence of three subpopulations, basically consistent with cluster analysis based on genetic distance [53]. Breseghello and Sorrells (2006) analyzed the population structure of 95 soft winter wheat cultivars of the eastern United States. These cultivars were divided into four subpopulations, which demonstrated that association mapping could complement and enhance previous QTL information for marker-assisted selection [54]. So, understanding the genetic diversity and structure populations of peanut cultivars would be vital to association mapping and molecular breeding program in peanut [55].

In the present study, the genetic diversity and population structure of 196 genotypes of peanut cultivar released in the last 5 decades from three ecological regions (the south region, the north region and the Yangtze River) in China were analyzed using 146 highly polymorphic SSR markers. Our objectives were to estimate the levels of genetic diversity, and to characterize the population structure of the Chinese peanut cultivars.

## Results

### Genetic diversity

The genotypes of 196 Chinese peanut cultivars (Table S1) were amplified using 210 SSR markers. Sixty-four SSR markers did not amplify in most of the accessions, and therefore were eliminated from the analysis. The remaining 146 SSR markers amplified 440 polymorphic bands with an average of 2.99, ranged from 2 to 9 per primer pair (Table 1; Table S2). The polymorphism information content (PIC) for the SSR loci ranged from 0.01 to 0.75, and the average PIC value was 0.38 (Table 1; Table S2). The gene diversity index ranged from 0.01 to 0.51, with an average of 0.11. Eighty-six rare alleles with a frequency less than 1% were identified at 66 loci in this study. Among these rare alleles, 13 rare alleles were found in cultivar ‘Nenghua 3’ and 10 rare alleles were found in ‘Jilinsilihong’ (Table 2, Table 3). Those two accessions were collected from Jilin province. The ratio of cultivars with rare alleles was the highest in cultivars from the south, and was the lowest in cultivars from the north. The 17 cultivars from Guangdong province had rare alleles, and no rare alleles were found in cultivars from Guizhou province (Table 2).

**Table 1:** Summary statistics of the 146 SSR markers used in this study.

	MAF	AN	GD	PIC
Max	0.99	9	0.51	0.75
Min	0.30	2	0.01	0.01
Mean	0.63	2.99	0.11	0.38

Notes: MAF, Major allele frequency; AN, Number of allele per locus; GD, Gene diversity; PIC, Polymorphism information content.

**Table 2:** Summary genetic statistics of peanut cultivars from different provinces in China.

Region	Province	NS	MAF	AN	GD	PIC	NRA
the South	Fujian	19	0.76	2.31	0.12	0.28	9
	Guangdong	30	0.78	2.50	0.12	0.27	17
	Guangxi	16	0.74	2.44	0.12	0.31	12
	Total	65	0.75	2.78	0.12	0.30	38
the Yangtze	Guizhou	3	0.79	1.53	0.17	0.18	0
River	Sichuan	10	0.77	2.07	0.15	0.26	5
	Hubei	16	0.68	2.41	0.14	0.34	8
	Hunan	3	0.76	1.76	0.12	0.24	2
	Total	32	0.67	2.59	0.14	0.35	15
the North	Jiangsu	12	0.74	2.26	0.16	0.30	8
	Anhui	5	0.76	1.9	0.13	0.26	2
	Hebei	9	0.79	2.04	0.15	0.24	4
	Henan	37	0.76	2.45	0.14	0.28	15
	Shandong	34	0.70	2.55	0.13	0.34	10
	Jilin	2	0.82	1.47	0.15	0.16	2
	Total	99	0.72	2.76	0.14	0.35	39

Notes: NS, Number of sample; MAF, Major allele frequency; AN, Number of allele per locus; GD, Gene diversity; PIC, Polymorphism information content; NRA, Number of cultivar with rare allele.

**Table 3:** Number of rare alleles in different cultivars.

No. of sample*	Name of sample	Number of rare alleles	Province
44	Nenghua 3	13	Jilin
43	Jilinsilihong	10	Jilin
3	Dapigu	4	Fujian
71	Shanyou 188	4	Guangdong
39	Shanyou 523	3	Guangdong
60	Yueyou 13	3	Guangdong
48	Zhongkaihua 2	3	Guangdong
26	Guihuahong 35	3	Guangxi
35	Guihua 166	3	Guangxi
95	Yuhua 14	3	Henan
114	Ehua 2	3	Hubei
143	Rudongwanerqing	3	Jiangsu
185	Hua 17	3	Shandong
135	Tianfu 18	3	Sichuan

Note: *No. of sample was in accord with no. of sample in Table S1.

The average number of alleles per locus was the greatest in cultivars from the south, and was the least in cultivars from the Yangtze River. The average gene diversity index was the lowest in cultivars from the south, followed by that from the north (Table 2). The average number of alleles per locus was increasing in the cultivars with each decade, but the ratio of cultivars with the rare allele was decreasing. In contrast, the parameters of the average gene diversity index and the frequency of major allele did not change much (Table S3).

### Genetic relationships

**Figure 1:**
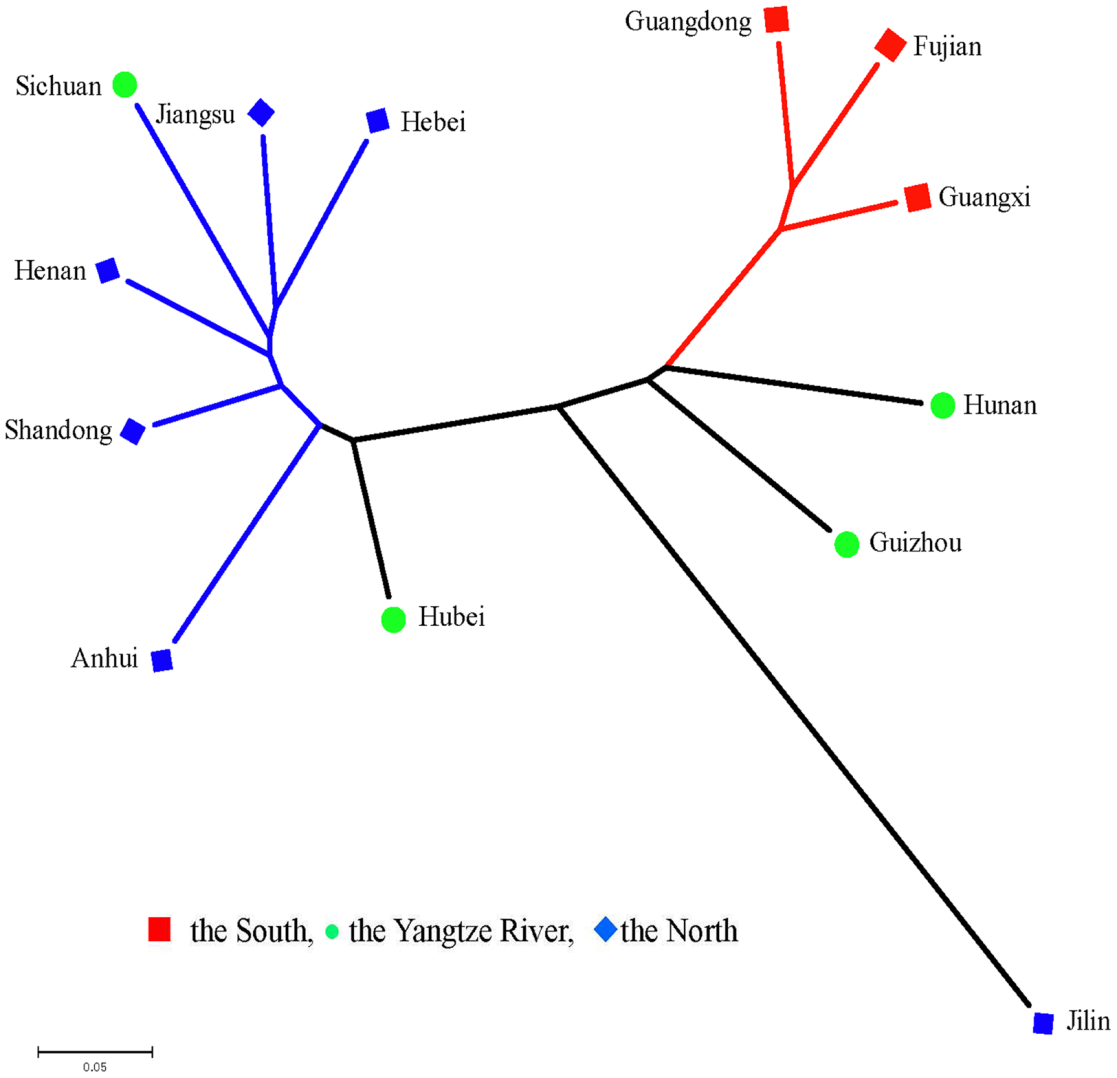
Unrooted neighbor joining tree of peanut cultivars from 13 provinces in China.

There was the largest genetic distance (0.40) in cultivars between the south and the north. Cultivars from the Yangtze River were very similar to those from the north (0.12). For different provinces, genetic distances between the populations from the south (Fujian, Guangdong, Guangxi) and the north (Shandong, Henan, Hebei, Jiangsu) were larger than those of others. Population of Shandong showed the smallest genetic distance with population of Henan (0.12), whereas population of Jilin showed the greatest genetic distance with populations of Anhui, Henan and Jiangsu (0. 55) (Table 4). Pairwise comparison on the basis of the values of F-Statistics (*F_st_*) could be interpreted as standardized population distances between two populations. The *F_st_* values would almost be in accordance with the genetic distances. A neighbor-joining tree of the thirteen province populations was constructed based on Nei’s genetic distances, showing significant difference between cultivars from the south and from the north (Fig. 1).

**Table 4:** Pairwise estimates of *F_st_* and Nei’s genetic distance based on 146 SSR markers among the 13 major peanut production provinces in China.

Regions	Provinces	I			II				III					
		Fujian	Guangdong	Guangxi	Guizhou	Sichuan	Hubei	Hunan	Jiangsu	Anhui	Hebei	Henan	Shandong	Jilin
	Fujian		0.04	0.02	0.14	0.44	0.24	0.09	0.40	0.3	0.43	0.41	0.3	0.35
the South (I)	Guangdong	0.14		0.03	0.13	0.46	0.26	0.13	0.41	0.31	0.44	0.41	0.32	0.4
	Guangxi	0.13	0.14		0.06	0.39	0.19	0.04	0.33	0.23	0.37	0.36	0.25	0.32
	Guizhou	0.28	0.26	0.26		0.35	0.07	0.00*	0.27	0.15	0.32	0.3	0.14	0.45
the Yangtze River (II)	Sichuan	0.51	0.51	0.47	0.43		0.11	0.34	0.06	0.12	0.11	0.09	0.08	0.45
	Hubei	0.34	0.35	0.31	0.28	0.25		0.08	0.08	0.03	0.09	0.07	0.01	0.29
	Hunan	0.26	0.27	0.25	0.21	0.45	0.30		0.28	0.15	0.33	0.31	0.15	0.29
	Jiangsu	0.48	0.47	0.43	0.38	0.19	0.21	0.42		0.06	0.01	0.06	0.04	0.4
the North (III)	Anhui	0.39	0.38	0.34	0.29	0.26	0.21	0.34	0.23		0.07	0.11	0.12	0.39
	Hebei	0.49	0.48	0.44	0.37	0.21	0.22	0.41	0.15	0.22		0.07	0.04	0.48
	Henan	0.46	0.46	0.43	0.38	0.18	0.17	0.42	0.14	0.24	0.17		0.03	0.45
	Shandong	0.39	0.39	0.35	0.32	0.20	0.13	0.34	0.16	0.21	0.17	0.12		0.34
	Jilin	0.44	0.45	0.45	0.50	0.19	0.50	0.47	0.55	0.55	0.54	0.55	0.16	

Note: Genetic distance estimates appear below the diagonal and pairwise *F_st_* appears above the diagonal. 0.00* stands for the data being less than 0.005.

According to Nei’s genetic distance based on 146 SSR loci in different years, cultivars released before 1970 had greatest Nei’s genetic distance. Among other each decade, Nei’s genetic distances did not change much (Table S4), which shows that the genetic diversity of peanut cultivated varieties had no obvious changes.

### Population structure

**Figure 2:**
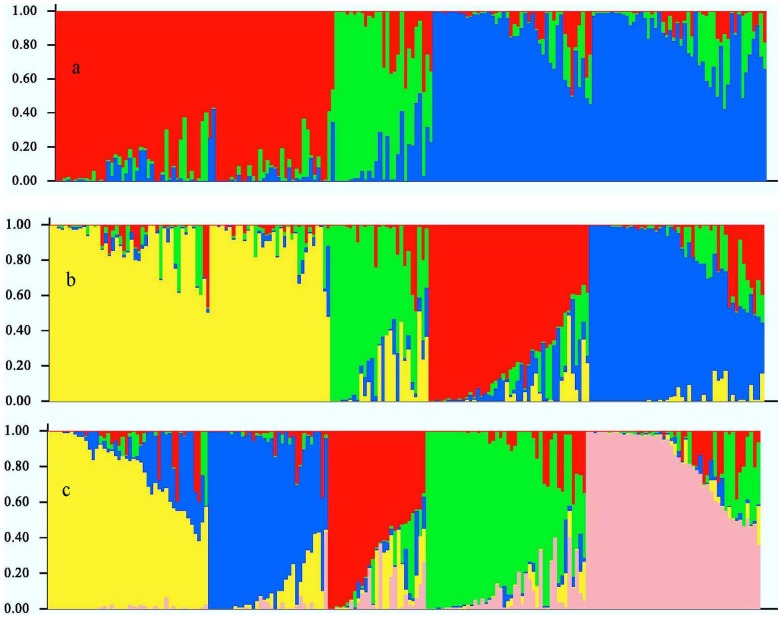
Membership probability of assigning genotypes of 196 peanut cultivated varieties to (a) three, (b) four, (c) five subgroups. The height of each bar represents the probability of a variety belonging to different subgroup. The varieties were sorted according to serial number of cultivated varieties (Table S1).

**Figure 3:**
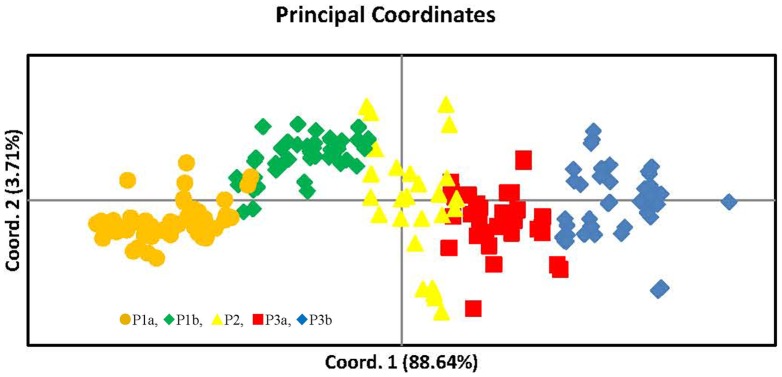
Principal coordinates analysis of five subpopulations of the 196 peanut cultivated varieties in China. Coord.1(88.64%) and Coord.2(3.71%) refer to the first and second principal component, respectively.

**Figure 4:**
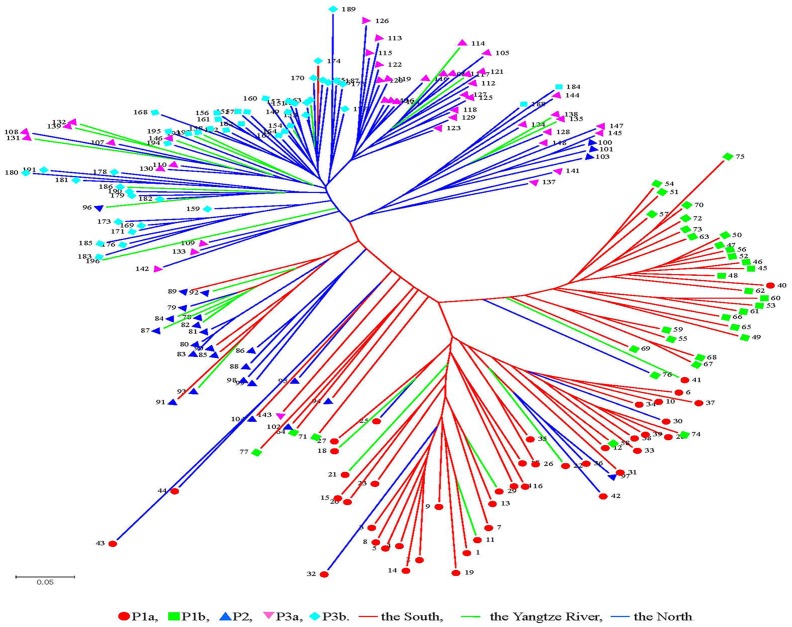
Unrooted neighbor-joining tree of 196 peanut cultivated varieties. The varieties were sorted according to serial number of cultivated varieties (Table S1).

The population structure of the Chinese peanut cultivars was inferred using Structure 2.0 software based on 146 SSR markers. The number of subpopulations (K) was identified based on maximum likelihood and delta K (▵*K*) values (Fig. S1). At K = 3, accessions were divided into three main populations, here denoted as P1, P2 and P3, respectively (Fig. 2a, Table 5). P1 contained 77 accessions, among which 64 were from the southern provinces including Guangdong, Guangxi and Fujian, 7 were from the northern provinces including Shandong, Henan and Jilin, and 6 were from the Yangtze Rivers including Hubei, Hunan and Guizhou provinces. The two cultivars from Jilin province were also clustered in P1. P2 had 27 accessions, most from Hubei, Henan and Shandong provinces. P3 contained 92 accessions, 75 of which were from the northern provinces including Shandong, Henan, Hebei, Jiangsu, Anhui, and 1 was from Guangxi province belonging to the south, and 16 cultivars were from the Yangtze River including 10 of Sichuan and 6 of Hubei (Table 5). At K =  4, two sub-subgroups (P1a-P1b) existed in P1, they consisted of 42 and 35 accessions, respectively (Fig. 2b, Table 5). The two cultivars from Jilin province were clustered in P1b. At K =  5, P3 could be divided into two sub-subgroups (P3a-P3b) which consisted of 44 and 48, respectively (Fig. 2c, Table 5). PCoA and PCA, which were based on marker genotypes, revealed five distinct clusters for the entire population that were related to their germplasm regions (Fig. 3, Fig. S2). The first and second principal coordinates explained 88.64% and 3.71% of the molecular variance, respectively. The first two principal components explained 66.46% and 8.08% of the molecular variance, respectively. Furthermore, neighbor-joining tree showed five branches within the peanut cultivars, which were fairly consistent with the structure-based membership assignment for most of the cultivars (Fig. 4).

**Table 5:** The accessions assigned to different populations by the software STRUCURE.

Regions	Province	P1		P2	P3		Total
		P1a	P1b		P3a	P3b	
the South	Fujian	9	10	0	0	0	19
	Guangdong	12	17	1	0	0	30
	Guangxi	10	5	0	0	1	16
	Total	31	32	1	0	1	65
the Yangtze	Guizhou	1	0	2	0	0	3
River	Hunan	2	0	1	0	0	3
	Hubei	3	0	7	3	3	16
	Sichuan	0	0	0	7	3	10
	Total	6	0	8	10	6	29
the North	Hebei	0	0	2	6	1	9
	Henan	1	0	4	6	26	37
	Jiangsu	0	0	0	11	1	12
	Shandong	3	1	9	8	13	34
	Jilin	0	2	0	0	0	2
	Anhui	1	0	1	3	0	5
	Total	5	3	16	34	41	99
Total		42	35	27	44	48	196

### Analysis of molecular variance

Released years, geographic origins and population structures provided the organizing data for hierarchical AMOVA. In the total genetic variance among populations based on the released years, 34% was attributed to the released year, and the remaining 66% was explained by individual differences within populations (Table 6). In the total genetic variance among populations of the geographic origins, 31% was attributed to the geographic origins, and the remaining 69% was explained by individual differences within populations (Table 6). In the total genetic variance among populations based on structure, 36% was attributed to the populations based on structure, and the remaining 64% was explained by individual differences within populations. These results demonstrated that different levels of genetic variance existed among released years, geographic origins, sub-groups, and individuals within populations, respectively.

**Table 6:** Analysis of molecular variance (AMOVA) in different populations.

Source of variation	d.f.	Sum of squares	Variance components	Percentage variation	p
Model-based population					
Among clusters	4	7793	48.41	36%	
Within	189	16487	87.24	64%	P<0.05
Geographic origin					
Among clusters	12	7787	39.95	31%	
Within	182	16493	90.62	69%	P<0.05
Released year					
Among clusters	4	6719	42.70	34%	
Within	189	17561	92.92	66%	P<0.05

## Discussion

### Comparing of peanut genetic diversity

SSR markers have been extensively used to detect the variability in peanut genotypes and to evaluate their genetic diversity. Compared with previous reports, the genetic diversity of the 196 Chinese peanut cultivars in this study was at a lower level, as reflected by the average number of alleles per locus (2.99) and the gene diversity index (0.11). Other researchers reported averages of 2.2 to 8.1 alleles per locus in various peanut collections [15]–[16], [20], [51]. A study of U.S. peanut mini-core collection uncovered an average of 8.1 alleles per locus [16], [51]. 168 accessions of peanut germplasm from 42 counties of five continents were employed for SSR analyses using 27 SSR markers, 4.29 alleles were identified per locus [16]. In Chinese core collection, polymorphic bands of var. *hypogaea* germplasm was 4.0, and breeding lines was 2.2 [15]. In this study, allele number per locus of cultivars would be similar to that of breeding lines [15], indicating that there would be less genetic diversity in released cultivars than landraces. In fact, the breeding lines and cultivars (outside of landrace) had the same origins from crossing.

In this study, gene diversity index was 0.11, which was lower than those of previous peanut collections (0.15–0.18) [16], [51]. A study of U.S. peanut mini-core collection uncovered an average of 0.15–0.18 gene diversity index in ssp. *hypogaea* and ssp. *fastigaea* [16], [51]. Although allelic diversity and gene diversity index could be used as indicators of genetic variation, such values were relative and depend on the number of polymorphic loci and the relatedness of genotypes analyzed. But in this study, allelic diversity and gene diversity index identified that there was a lower level of genetic diversity in Chinese peanut cultivars than landraces [14]–[15], which was consistent with the results reported in rice [44]–[45], [52], wheat [48], [53] and soybean [50], [54]. Thus, it was necessary to introduce more peanut germplasm into Chinese peanut cultivars to broaden their genetic diversity.

Some of the SSR markers generated genotype-specific bands, which could be used as molecular identity data for specific genotypes. Eighty-six rare alleles with a frequency less than 1% were identified in 196 genotypes peanut cultivars using 146 SSR markers. There was 0.45 in average rare alleles per SSR marker, which was lower than that of US mini-core collection (1.68) [16], [51]. This finding also indicated there would be low genetic diversity in Chinese peanut cultivars. Among these rare alleles, 13 unique rare alleles were found in the accession ‘Nenghua 3’, 10 were found in the accession ‘Jilinsilihong’. These results indicated that the two cultivars are distinct from the others, probably due to that the two cultivars belong to ssp. *fastigiata* var. *fastigiata* that is rarely planted in China. Many unique rare alleles would be discovered in ssp. *fastigiata*, showing that ssp. *fastigiata* would have more genotype-specific bands than ssp. *hypogaea* do [16].

### Analysis of genetic relationships of peanut cultivars

On the basis of geography origin of different peanut cultivars, peanut cultivars were divided in the three ecological regions: the south region, the north region and the Yangtze River region, which are the three ecological regions of the national peanut regional test in China [2]. The south includes Fujian, Guangxi, Guangdong provinces etc. The Yangtze River region includes Hunan, Hubei, Sichuan, Guizhou provinces etc. The north includes Shandong, Henan, Hebei, Jiangsu, Anhui, Jilin provinces et al. There is the largest denetic distance in cultivars between the south and the north. Cultivars from the Yangtze River are very similar to those from the north. In fact, many cultivars were divided into P3 population using structure analysis (Table 5), indicating that the cultivars from the Yangtze River and those from the south are different. Genetic distances between populations from the south and the north were larger than those of between other populations, which indicate that there are obvious differences between the south populations and the north.

All populations from the south were clustered together, and most of the cultivars were ssp. *fastigiata.* Similarly, most of populations from the north were clustered together, and most of the cultivars were ssp. *hypogaea* [1]–[2]. Consistently, the US peanut mini-core collections were grouped into two major clusters corresponding to the subspecies groupings, i.e. subspecies *fastigiata* and *hypogaea* [16]. Although Jilin lies in the north, the cultivars ‘Nenghua 3’ and ‘Jilinsilihong’ from Jinlin province are ssp. *fastigiata* var. *fastigiata*, which were clustered with those from the south together. AMOVA analysis revealed that, in the total genetic variance of the geographic origins, 31% were attributed to the geographic origins (Table 6), which indicated that there were obvious differences in peanut cultivars from different provinces in China.

Our results showed that the average alleles per marker increased in released cultivars each decade, while there are no obvious differences for gene diversity and major allele frequency in released cultivars (Table S3). The cultivars released before the 1970s had greater pairwise estimates of *F_st_* and Nei’s genetic distance than those of the other years, and they did not change much after the 1970s (Table S4). These results showed that the genetic diversity of peanut cultivars had no obvious change since then. The reason might be that early cultivars in this study were landraces and main parents, which did not suffer from reciprocal cross on a large scale, and they presented a higher genetic diversity consequentially. Many early accessions were introduced into cross breeding, which led to an increase of the average alleles per marker. However, strong artificial selection generated similar derivatives as the parents, and then these derivatives had little genetic diversity. According to AMOVA, in the total genetic variance of the released years, 66% was explained by individual differences within populations (Table 6). These results showed that there are obvious differences in peanut cultivars released in different decade in China.

### Population structure of peanut cultivars in China

To identify the true optimal number of subsets (K) in STRUCTURE, LnP(D) and *ΔK* were chosen [44]–[49], [55]. The K value that provides the maximum likelihood, called LnP(D) in STRUCTURE, is generally considered as the optimal number of subdivisions [41]. In this study, the distribution of L(K) did not show a clear mode for the true K in China peanut cultivars (Fig. S2b). Thus, another ad hoc quantity (*ΔK*) was used to overcome the difficulty for interpreting the real K values [56]. The highest value of *ΔK* for the 196 peanut cultivars is K  =  5 (Fig S2a). Clustering bar plots with K  =  3–5 are shown in Figure 2. At K  = 3, all 196 accessions are divided into three subpopulations from the south, the north and the Yangtze River, respectively, which confirmed the above results (Fig. 1, Table 4). At K =  4, there are two sub-subgroups (P1a-P1b), which consist of 42 and 35 accessions, respectively (Table 4), showing that there was genetic differentiation in the cultivars from the south. At K =  5, two sub-subgroups existed in the P3 (P3a-P3b) which consisted of 44, 48, respectively (Table 4), indicating that there was genetic differentiation in the cultivars from the north. Analysis of these data identified the major substructure groups when the number of populations was set at three with the highest value of *ΔK* (Fig. 2), which was consistent with the clustering results based on genetic distance (Fig. 4). As shown in Figure 2, most of the accessions were clearly classified into one of the five subpopulations. PCoA and PCA based on the marker genotypes revealed five distinct clusters for the entire population (Fig. 3, Fig. S2), which were related to their germplasm regions. Furthermore, the neighbor-joining tree showed five branches within the peanut cultivars, which was fairly consistent with the structure-based membership assignment for most of the cultivars (Fig. 4). Cultivars of Sichuan province were divided into P3a and P3b populations, which were caused by the cultivars related to ssp. *hypogaea* ‘Luojiangjiwo’ [1], while most cultivars from the north are related to ssp. *hypogaea* [1]–[2].

A narrow genetic base had been reported for peanut cultivars in China, probably because of high selection pressure for good grain quality and repeated use of the same-origin parents with proven yield ability in the breeding program, resulting in significant genetic erosion of the local peanut gene pool [1]–[2]. The results presented in this study suggested that the genetic base of Chinese peanut cultivars be relatively narrow, and a wider range of accessions should be introduced to improve present varieties in future breeding programs. In fact, according to the result of population structure, there were very abundant genetic differentiations in cultivars of the south or the north. The cultivars adapted to local ecological environment, which were the best parents for breeding. Our results suggested that there was highest possible to improve the yield by crossing in cultivars from between P1a and P1b in the south or from between P3a and P3b in the north.

## Materials and Methods

### Plant material

According to the information of Peanut varieties and their pedigree in China [1], and Peanut Genetics and Breeding in China [2], a total of 196 accessions of cultivated varieties were collected from 13 different provinces of China (Table S1), these accessions were planted at Oil Crops Research Institute, Chinese Academy of Agriculture Sciences.

### DNA extraction

Two or three young unopened leaves were collected from each accession, and used for DNA extraction using the hexadecyl trimenthyl ammonium bromide (CTAB) method [57]. DNA was quantified with a Beckman DU-650 spectrophotometer. Integrity and quality of DNA was evaluated by electrophoresis on a 1% agarose gel.

### SSR array

The SSR primer sequences were selected based on the previous reports [17]–[18], [25]–[28], [31], [33]–[46], and synthesized by BGI (Table S2). The 10 μL PCR reaction mixture consisted of 0.4 mM dNTPs, 0.3 pM primers, 2.0 mM Mg_2_^+^, 1× Taq buffer, 0.5U Taq DNA polymerase (Fermentas) and 15 ng DNA. Amplification was carried out using a PTC-100 Thermocycler (MJ Research). The amplified products were visualized by 6% denaturing polyacrylamide gel (PAGE) followed by silver staining as described by Ren [58].

### Data analysis

The polymorphism bands were recorded as 1 (present) or 0 (absent) for the same amplified fragments. Data format could be changed according to different analysis software. Major allele frequency, number of alleles per locus, gene diversity and polymorphism information content (PIC) were analyzed using PowerMarker V3.25 [46]. Genetic distance (GD) and pairwise values of F-Statistics (*F_st_*) between populations were calculated using PowerMarker V3.25. The dendrogram was constructed based on genetic distance calculated using the Neighbour subroutine of PoweMarker V3.25 as described [59].

based program Structure [41]. The number of subpopulations (*k*) was assumed to be from 1 to 10, without admixture and with correlated allele frequencies, and the burn-in time and iterations for each run were both set to 50,000. Ten replications were used for each *k*. Due to the difficulties associated with finding the highest posterior probability before a large *k* value was examined, LnP(D) and Evanno’s Δ*k* [56] were used to determine the most appropriate *k* value. It is calculated as Δ*k*  =  *M*[|L(k – 1) – 2L(*k*) + L(*k* + 1)|]/*S*[L(*k*)], in which L(*k*) represents the *k*th LnP(D), M is the mean of 10 runs, and S is their standard deviation.

Principal components analysis (PCA) was a visualization technique commonly used in multivariate statistics, which also identifies eigenvectors and amounts of variance and cumulatively explained variances per component. The PCA analysis was conducted using the NTSYS package [42]. Genetic similarities between genotypes in each group were determined with a coefficient based on the proportion of shared alleles and on a principal coordinates analysis (PCoA) using the software package GenAleX [43]. The molecular variance of subgroups, provinces and years were calculated using an analysis of molecular variance (AMOVA) approach in the software package Arlequin [60].

## Supporting Information

Figure S1 **The modal value of this distribution is the true** ***K*** **. The** ***Δk*** **of 10 repeats based on STRUCTURE calculation using SSR data.** ***Δk*** **calculated as** ***Δk*** **=** ***m*** **(** ***| L*** **(** ***K+1*** **)** ***−2 L*** **(** ***K*** **)** ***+ L*** **(** ***K−1)|*** **)/** ***S*** **[** ***L*** **(** ***K*** **)].**
(DOC)

Figure S2 **Principal Component Analysis of five subpopulations of the 196 peanut cultivated varieties in China.**
(DOC)

Table S1 **Accessions, variety names, origin, region, released year of 196 peanut cultivars in China.**
(DOC)

Table S2 **Summary statistics of the 146 SSR markers used in this study.** MAF: major allele frequency, AN: number of alleles per locus, GD: gene diversity, PIC: polymorphism information content.
(DOC)

Table S3 **Summary genetic statistics of peanut cultivars in different years.**
(DOC)

Table S4 **Pairwise estimates of** ***Fst*** **and Nei’s genetic distance based on 146 SSR loci among different years.**
(DOC)
