# Supplementary material for: Genetic Diversity and Population Structure of the Major Peanut (Arachis hypogaea L.) Cultivars Grown in China by SSR Markers
Source: PLoS One. 2014 Feb 10;9(2):e88091. doi: 10.1371/journal.pone.0088091 (PMC3919752; doi:10.1371/journal.pone.0088091)
Supplement: Figure S1 — The modal value of this distribution is the true K . The Δk of 10 repeats based on STRUCTURE calculation using SSR data. Δk calculated as Δk = m ( | L ( K+1 ) −2 L ( K ) + L ( K−1)| )/ S [ L ( K )]. (DOC) [file pone.0088091.s001.doc]

**Figure S1**


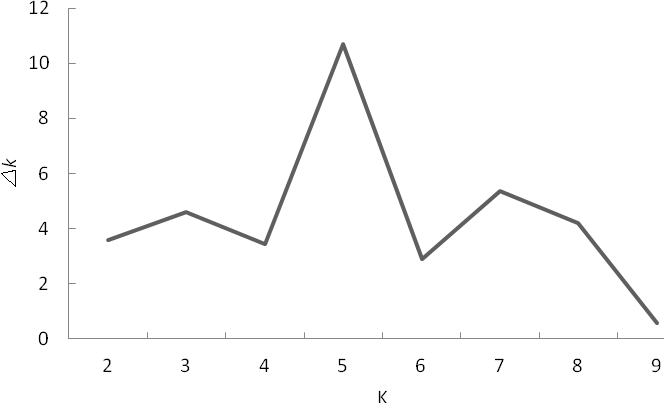

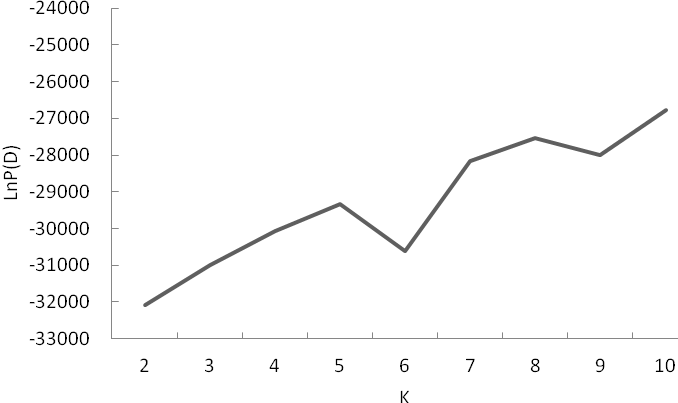


B

A

**Figure S1. The modal value of the distribution is the true *K*.** The *Δk* of 10 repeats based on STRUCTURE calculation using SSR data. *Δk* calculated as *Δk* = *m* (*| L* (*K+1*) *−2 L* (*K*) *+ L* (*K−1)|*) / *s* [*L* (*K*)]*.*
