# Supplementary material for: Genetic Diversity and Population Structure of the Major Peanut (Arachis hypogaea L.) Cultivars Grown in China by SSR Markers
Source: PLoS One. 2014 Feb 10;9(2):e88091. doi: 10.1371/journal.pone.0088091 (PMC3919752; doi:10.1371/journal.pone.0088091)
Supplement: Figure S2 — Principal Component Analysis of five subpopulations of the 196 peanut cultivated varieties in China. (DOC) [file pone.0088091.s002.doc]

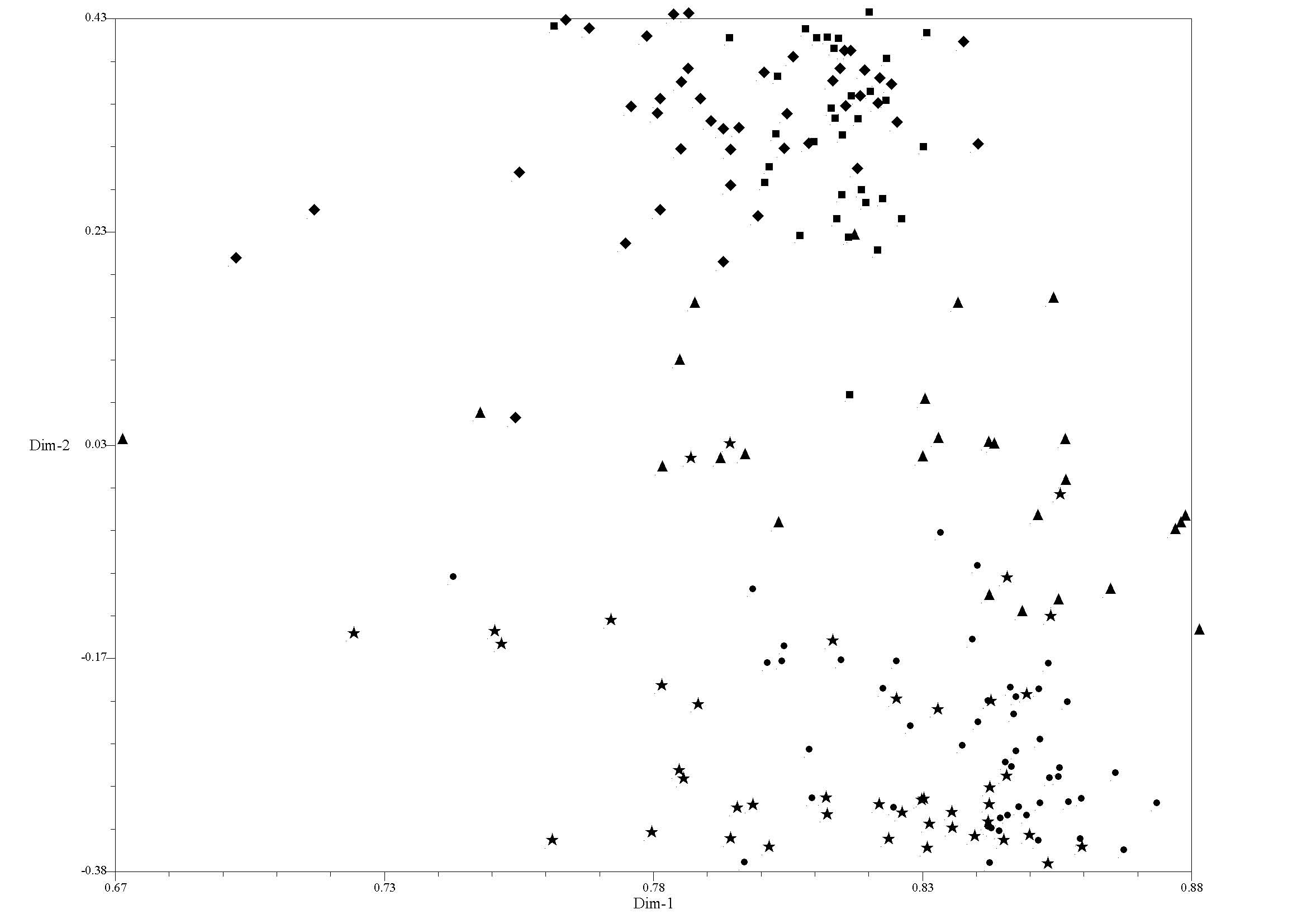


◆1,■2,▲3,★4,●5

**Figure S2. Principal component analysis of five subpopulations of the 196 peanut cultivated varieties in China.** PCA1(66.46%) and PCA2(8.08%) refer to the first and second principal component, respectively.
