# Supplementary material for: Genetic Diversity and Population Structure of the Major Peanut (Arachis hypogaea L.) Cultivars Grown in China by SSR Markers
Source: PLoS One. 2014 Feb 10;9(2):e88091. doi: 10.1371/journal.pone.0088091 (PMC3919752; doi:10.1371/journal.pone.0088091)
Supplement: Table S1 — Accessions, variety names, origin, region, released year of 196 peanut cultivars in China. (DOC) [file pone.0088091.s003.doc]

**Table S1 Accessions, variety names, province, origin, released year of 196 peanut cultivars in China.**

| No. | Name | Province | Origin | Type | Released year |
| --- | --- | --- | --- | --- | --- |
| 1 | Sanjiagong | Fujian | The south | Landrace | 1959 |
| 2 | Heyue 1 | Guangxi | The south | Line | 1964 |
| 3 | Dapigu | Fujian | The south | Landrace | 1958 |
| 4 | Fenghong | Guangdong | The south | Line | 1970 |
| 5 | Heyue 2 | Guangxi | The south | Line | 1964 |
| 6 | Quanhua327 | Fujian | The south | Line | 2001 |
| 7 | Guihuahong 95 | Guangxi | The south | Line | 2008 |
| 8 | Xiaoliuqiu | Fujian | The south | Landrace | 1958 |
| 9 | Bairizi | Guangdong | The south | Landrace | 1959 |
| 10 | Puhua 1 | Fujian | The south | Line | 2009 |
| 11 | Xianghuasheng 1 | Hunan | The Yangtze River | Line | 1985 |
| 12 | Heyou 77 | Guangxi | The south | Line | 1975 |
| 13 | Yueyou 92 | Guangdong | The south | Line | 1986 |
| 14 | Dajieren | Fujian | The south | Landrace | 1959 |
| 15 | Furonghuasheng | Hunan | The Yangtze River | Landrace | 1984 |
| 16 | Guihua 22 | Guangxi | The south | Line | 2000 |
| 17 | Guihua 17 | Guangxi | The south | Line | 1995 |
| 18 | Hongmeizao | Hubei | The Yangtze River | Landrace | 1984 |
| 19 | Puyou 3 | Fujian | The south | Line | 1978 |
| 20 | Taishanzhenzhu | Guangdong | The south | Landrace | 1978 |
| 21 | Zhongliuqiu | Fujian | The south | Landrace | 1958 |
| 22 | Ehua 4 | Hubei | The Yangtze River | Line | 1987 |
| 23 | Shinan 3 | Guangdong | The south | Line | 1979 |
| 24 | Wuyou 1 | Guangxi | The south | Line | 1976 |
| 25 | Haihua 1 | Shandong | The north | Line | 1984 |
| 26 | Guihuahong 35 | Guangxi | The south | Line | 2008 |
| 27 | Shitouqi | Guangdong | The south | Landrace | 1957 |
| 28 | Yueyou 256-2 | Guangdong | The south | Line | 1991 |
| 29 | Qianhuasheng 1 | Guizhou | The Yangtze River | Line | 2000 |
| 30 | Wanhua 4 | Anhui | The north | Line | 2006 |
| 31 | Fuhua 4 | Fujian | The south | Line | 2010 |
| 32 | Hua 17 | Shandong | The north | Line | 1982 |
| 33 | Yueyou 551 | Guangdong | The south | Line | 1978 |
| 34 | Guihua 836 | Guangxi | The south | Line | 2010 |
| 35 | Guihuahong 166 | Guangxi | The south | Line | 2008 |
| 36 | Yuhua 12 | Henan | The north | Line | 1999 |
| 37 | Zhanyou 55 | Guangdong | The south | Line | 2006 |
| 38 | Shanyou 199 | Guangdong | The south | Line | 2008 |
| 39 | Shanyou 523 | Guangdong | The south | Line | 1991 |
| 40 | Yueyou 116 | Guangdong | The south | Line | 1982 |
| 41 | Zhonghua 2 | Hubei | The Yangtze River | Line | 1990 |
| 42 | Luhua 12 | Shandong | The north | Line | 1994 |
| 43 | Jilinsilihong | Jilin | The north | Landrace | 1970 |
| 44 | Nenghua 3 | Jilin | The north | Line | 2009 |
| 45 | Zhongkaihua 1 | Guangdong | The south | Line | 2006 |
| 46 | Yueyou 52 | Guangdong | The south | Line | 2010 |
| 47 | Minhua 5 | Fujian | The south | Line | 2006 |
| 48 | Zhongkaihua 2 | Guangdong | The south | Line | 2007 |
| 49 | Yueyou 7 | Guangdong | The south | Line | 2004 |
| 50 | Zhanyou 62 | Guangdong | The south | Line | 2003 |
| 51 | Heyou 11 | Guangxi | The south | Line | 2010 |
| 52 | Yueyou 79 | Guangdong | The south | Line | 2002 |
| 53 | Shanyou 27 | Guangdong | The south | Line | 1997 |
| 54 | Heyou 10 | Guangxi | The south | Line | 2010 |
| 55 | Fuhua 3 | Fujian | The south | Line | 2010 |
| 56 | Minhua 8 | Fujian | The south | Line | 2008 |
| 57 | Heyou 12 | Guangxi | The south | Line | 2010 |
| 58 | Zhanyou75 | Guangdong | The south | Line | 2008 |
| 59 | Fuhua 6 | Fujian | The south | Line | 2010 |
| 60 | Yueyou 13 | Guangdong | The south | Line | 2006 |
| 61 | Yueyou 223 | Guangdong | The south | Line | 1992 |
| 62 | Zhenzhuhong 1 | Guangdong | The south | Line | 2002 |
| 63 | Fuhua 5 | Fujian | The south | Line | 2003 |
| 64 | Quanhua 646 | Fujian | The south | Line | 2000 |
| 65 | Yueyou 9 | Guangdong | The south | Line | 2004 |
| 66 | Yueyou 40 | Guangdong | The south | Line | 2008 |
| 67 | Wuyou 7 | Guangxi | The south | Line | 2000 |
| 68 | Wuyou 4 | Guangxi | The south | Line | 1982 |
| 69 | Fuhua 8 | Fujian | The south | Line | 2010 |
| 70 | Quanhua 6 | Fujian | The south | Line | 1997 |
| 71 | Shanyou 188 | Guangdong | The south | Line | 2008 |
| 72 | Kanghuang 1 | Guangdong | The south | Line | 2003 |
| 73 | Quanhua 8 | Fujian | The south | Line | 2006 |
| 74 | Yueyou 20 | Guangdong | The south | Line | 2004 |
| 75 | Quanhua 10 | Fujian | The south | Line | 1995 |
| 76 | Huayu 20 | Shandong | The north | Line | 2002 |
| 77 | Shiyouhong 4 | Guangdong | The south | Line | 1978 |
| 78 | Zhonghua 8 | Hubei | The Yangtze River | Line | 2002 |
| 79 | Luhua 8 | Shandong | The north | Line | 1988 |
| 80 | Shanhua 10 | Shandong | The north | Line | 2009 |
| 81 | Fenghua 6 | Shandong | The north | Line | 2005 |
| 82 | Zhonghua 5 | Hubei | The Yangtze River | Line | 1998 |
| 83 | Shanhua 8 | Shandong | The north | Line | 2007 |
| 84 | Xianghua 2008 | Hunan | The Yangtze River | Line | 2008 |
| 85 | Qianhuasheng 2 | Guizhou | The Yangtze River | Line | 2006 |
| 86 | Yuanza 9307 | Henan | The north | Line | 2002 |
| 87 | Zhonghua 10 | Hubei | The Yangtze River | Line | 2004 |
| 88 | Yuanza 9102 | Henan | The north | Line | 2002 |
| 89 | Huayu 19 | Shandong | The north | Line | 2002 |
| 90 | Weihua 10 | Shandong | The north | Line | 2006 |
| 91 | Baisha 1016 | Guangdong | The south | Line | 1978 |
| 92 | Zhonghua 16 | Hubei | The Yangtze River | Line | 2009 |
| 93 | Qianhuasheng 4 | Guizhou | The Yangtze River | Line | 2006 |
| 94 | Kainong 8598 | Henan | The north | Line | 2002 |
| 95 | Yuhua 14 | Henan | The north | Line | 2002 |
| 96 | Zhonghua 15 | Hubei | The Yangtze River | Line | 2008 |
| 97 | Hua 11 | Shandong | The north | Line | 1983 |
| 98 | Kangqing 10 | Shandong | The north | Line | 1980 |
| 99 | Luhua 3 | Shandong | The north | Line | 1984 |
| 100 | Wanhua 7 | Anhui | The north | Line | 2008 |
| 101 | Jihua 6 | Hebei | The north | Line | 2010 |
| 102 | Zhonghua 6 | Hubei | The Yangtze River | Line | 2000 |
| 103 | Jihua 7 | Hebei | The north | Line | 2009 |
| 104 | Zhonghua 4 | Hubei | The Yangtze River | Line | 1995 |
| 105 | Xuzhou 402 | Jiangsu | The north | Line | 1954 |
| 106 | Shanhua 7 | Shandong | The north | Line | 2007 |
| 107 | Yuhua 4 | Henan | The north | Line | 1991 |
| 108 | Suiningerwo | Jiangsu | The north | Landrace | 1954 |
| 109 | 8130 | Shandong | The north | Line | 1993 |
| 110 | Wanhua 8 | Anhui | The north | Line | 2009 |
| 111 | Yuhua 16 | Henan | The north | Line | 2000 |
| 112 | Taihua 4 | Jiangsu | The north | Line | 2004 |
| 113 | Yuhua 9 | Henan | The north | Line | 1999 |
| 114 | Ehua 2 | Hubei | The Yangtze River | Line | 1984 |
| 115 | Hua 31 | Shandong | The north | Line | 1978 |
| 116 | Tangyou 4 | Hebei | The north | Line | 1978 |
| 117 | Tianfu 15 | Sichuan | The Yangtze River | Line | 2002 |
| 118 | Hua 37 | Shandong | The north | Line | 1983 |
| 119 | Shanhua 9 | Shandong | The north | Line | 2009 |
| 120 | Fenghua 5 | Shandong | The north | Line | 2005 |
| 121 | Pukehua 1 | Henan | The north | Line | 2003 |
| 122 | Xucaihua 1 | Jiangsu | The north | Line | 2006 |
| 123 | Xuxi 1 | Jiangsu | The north | Line | 1978 |
| 124 | Jiyou 6 | Hebei | The north | Line | 1988 |
| 125 | Ehua 3 | Hubei | The Yangtze River | Line | 1984 |
| 126 | Jiyou 4 | Hebei | The north | Line | 1986 |
| 127 | Taihua 3 | Jiangsu | The north | Line | 2002 |
| 128 | Tianfu 9 | Sichuan | The Yangtze River | Line | 1992 |
| 129 | Xuhzou 68-4 | Jiangsu | The north | Line | 1978 |
| 130 | Yuhua 8 | Henan | The north | Line | 1996 |
| 131 | Nanchonghunxuan 1 | Sichuan | The Yangtze River | Landrace | 1987 |
| 132 | Honganzhili | Hubei | The Yangtze River | Landrace | 1955 |
| 133 | Xuhua 4 | Jiangsu | The north | Line | 1989 |
| 134 | Fuhuasheng | Shandong | The north | Landrace | 1944 |
| 135 | Tianfu 18 | Sichuan | The Yangtze River | Line | 2005 |
| 136 | Jiyou 5 | Hebei | The north | Line | 1988 |
| 137 | kainong 8 | Henan | The north | Line | 1982 |
| 138 | Tianfu 14 | Sichuan | The Yangtze River | Line | 2001 |
| 139 | Tianfu 3 | Sichuan | The Yangtze River | Line | 1985 |
| 140 | Jiyou 2 | Hebei | The north | Line | 1975 |
| 141 | Wanhua 5 | Anhui | The north | Line | 2002 |
| 142 | Donghua 2 | Jiangsu | The north | Line | 1991 |
| 143 | Rudongwanerqing | Jiangsu | The north | Landrace | 1955 |
| 144 | Weihua 6 | Shandong | The north | Line | 2001 |
| 145 | Wanhua 6 | Anhui | The north | Line | 2007 |
| 146 | Tianfu 7 | Sichuan | The Yangtze River | Line | 1990 |
| 147 | Taihua 6 | Jiangsu | The north | Line | 2007 |
| 148 | Tanghua 10 | Hebei | The north | Line | 2009 |
| 149 | Hua 28 | Shandong | The north | Line | 1982 |
| 150 | Yuhua 1 | Henan | The north | Line | 1985 |
| 151 | Kainongbai 2 | Henan | The north | Line | 2006 |
| 152 | Yuhua 7 | Henan | The north | Line | 2000 |
| 153 | Luhua 1 | Shandong | The north | Line | 1983 |
| 154 | Zhonghua 1 | Hubei | The Yangtze River | Line | 1989 |
| 155 | Yuhua 9840 | Henan | The north | Line | 2007 |
| 156 | Kainong 36 | Henan | The north | Line | 2002 |
| 157 | Fenghua 1 | Shandong | The north | Line | 2001 |
| 158 | Kai 51-2 | Henan | The north | Line | 2008 |
| 159 | Luhua 11 | Shandong | The north | Line | 1992 |
| 160 | Luhua 9 | Shandong | The north | Line | 1988 |
| 161 | Kainong 41 | Henan | The north | Line | 2005 |
| 162 | Kainong 37 | Henan | The north | Line | 2003 |
| 163 | Tianfu 4 | Sichuan | The Yangtze River | Line | 1985 |
| 164 | Yuhua 6 | Henan | The north | Line | 1993 |
| 165 | Yuhua 2 | Henan | The north | Line | 1988 |
| 166 | Kainong 30 | Henan | The north | Line | 2001 |
| 167 | Yuhua 5 | Henan | The north | Line | 1993 |
| 168 | Yuhua 9331 | Henan | The north | Line | 2004 |
| 169 | Kainong 49 | Henan | The north | Line | 2007 |
| 170 | Hua 27 | Shandong | The north | Line | 1977 |
| 171 | Kainong 53 | Henan | The north | Line | 2008 |
| 172 | Luhua 4 | Shandong | The north | Line | 1985 |
| 173 | Kainong 60 | Henan | The north | Line | 2010 |
| 174 | Te 21 | Guangxi | The south | Line | 1995 |
| 175 | Yuhua 3 | Henan | The north | Line | 1992 |
| 176 | Huayu 22 | Shandong | The north | Line | 2003 |
| 177 | Jihua 4 | Hebei | The north | Line | 2006 |
| 178 | Yuhua 9717 | Henan | The north | Line | 2009 |
| 179 | Yuhua 15 | Henan | The north | Line | 2001 |
| 180 | Yuhua 9502 | Henan | The north | Line | 2007 |
| 181 | Yuhua 9620 | Henan | The north | Line | 2008 |
| 182 | Yuhua 9326 | Henan | The north | Line | 2005 |
| 183 | Huayu 16 | Shandong | The north | Line | 1999 |
| 184 | Xuhua 5 | Jiangsu | The north | Line | 1993 |
| 185 | Huayu 17 | Shandong | The north | Line | 1999 |
| 186 | Zhonghua 9 | Hubei | The Yangtze River | Line | 2004 |
| 187 | Yuhua 10 | Henan | The north | Line | 2000 |
| 188 | Weihua 8 | Shandong | The north | Line | 2003 |
| 189 | Luhua 14 | Shandong | The north | Line | 1995 |
| 190 | Yuhua 9327 | Henan | The north | Line | 2003 |
| 191 | Kai H03-3 | Henan | The north | Line | 2007 |
| 192 | Tianfu 8 | Sichuan | The Yangtze River | Line | 1989 |
| 193 | Luojiangjiwo | Sichuan | The Yangtze River | Landrace | 1954 |
| 194 | Jianggezhuangbanman | Shandong | The north | Landrace | 1954 |
| 195 | Puhua 17 | Henan | The north | Line | 2002 |
| 196 | Zhonghua 12 | Hubei | The Yangtze River | Line | 2006 |
