# Supplementary material for: Genetic Diversity and Population Structure of the Major Peanut (Arachis hypogaea L.) Cultivars Grown in China by SSR Markers
Source: PLoS One. 2014 Feb 10;9(2):e88091. doi: 10.1371/journal.pone.0088091 (PMC3919752; doi:10.1371/journal.pone.0088091)
Supplement: Table S2 — Summary statistics of the 146 SSR markers used in this study. MAF: major allele frequency, AN: number of alleles per locus, GD: gene diversity, PIC: polymorphism information content. (DOC) [file pone.0088091.s004.doc]

**Table S2.** Summary statistics of the 146 SSR markers used in this study. MAF, Major allele frequency; AN, Number of alleles per locus; GD, Gene diversity; PIC, Polymorphism information content.

| **Markers** | **MAF** | **AN** | **GD** | **PIC** | **Reserences** |
| --- | --- | --- | --- | --- | --- |
| AC1C11 | 0.58 | 2 | 0.01 | 0.37 | Moretzsohn et al., 2005 |
| AC2C5 | 0.56 | 3 | 0.16 | 0.40 | Moretzsohn et al., 2005 |
| AD91022 | 0.82 | 2 | 0.02 | 0.25 | Leal-Bertioli et al., 2009 |
| Ah193 | 0.58 | 2 | 0.01 | 0.37 | Moretzsohn et al., 2004 |
| AHBGST1003B4 | 0.68 | 3 | 0.12 | 0.39 | Moretzsohn et al., 2009 |
| AHGS0599 | 0.37 | 6 | 0.10 | 0.72 | Wang et al., 2012 |
| AHS2037 | 0.57 | 2 | 0.07 | 0.37 | Wang et al., 2012 |
| Ai119F10 | 0.30 | 6 | 0.04 | 0.69 | Leal-Bertioli et al., 2009 |
| ARS370 | 0.53 | 3 | 0.14 | 0.38 | Qin et al., 2012 |
| ARS120 | 0.51 | 3 | 0.04 | 0.51 | Qin et al., 2012 |
| ARS141 | 0.67 | 2 | 0.05 | 0.35 | Qin et al., 2012 |
| ARS173 | 0.53 | 2 | 0.39 | 0.37 | Qin et al., 2012 |
| ARS205 | 0.58 | 3 | 0.08 | 0.47 | Qin et al., 2012 |
| ARS214 | 0.41 | 4 | 0.29 | 0.66 | Qin et al., 2012 |
| ARS298 | 0.50 | 2 | 0.05 | 0.38 | Qin et al., 2012 |
| ARS535 | 0.52 | 2 | 0.19 | 0.37 | Qin et al., 2012 |
| ARS590 | 0.60 | 3 | 0.11 | 0.49 | Qin et al., 2012 |
| ARS627 | 0.43 | 6 | 0.11 | 0.60 | Qin et al., 2012 |
| ARS660 | 0.71 | 2 | 0.10 | 0.33 | Qin et al., 2012 |
| ARS715 | 0.75 | 4 | 0.02 | 0.37 | Qin et al., 2012 |
| ARS730 | 0.53 | 3 | 0.03 | 0.49 | Qin et al., 2012 |
| ARS731 | 0.50 | 5 | 0.05 | 0.60 | Qin et al., 2012 |
| ARS737 | 0.97 | 3 | 0.01 | 0.06 | Qin et al., 2012 |
| ARS742 | 0.53 | 4 | 0.07 | 0.51 | Qin et al., 2012 |
| ARS749 | 0.90 | 5 | 0.01 | 0.18 | Qin et al., 2012 |
| ARS751 | 0.62 | 3 | 0.01 | 0.39 | Qin et al., 2012 |
| ARS758 | 0.88 | 2 | 0.01 | 0.18 | Qin et al., 2012 |
| ARS771 | 0.62 | 3 | 0.05 | 0.37 | Qin et al., 2012 |
| ARS785 | 0.51 | 3 | 0.13 | 0.55 | Qin et al., 2012 |
| ARS797 | 0.61 | 3 | 0.01 | 0.38 | Qin et al., 2012 |
| ARS808 | 0.52 | 3 | 0.01 | 0.43 | Qin et al., 2012 |
| EE16 | 0.55 | 2 | 0.01 | 0.37 | Liang et al., 2009 |
| EE22 | 0.62 | 2 | 0.13 | 0.36 | Liang et al., 2009 |
| EM132 | 0.50 | 4 | 0.33 | 0.49 | Liang et al., 2009 |
| EM28 | 0.77 | 3 | 0.01 | 0.31 | Liang et al., 2009 |
| EM59 | 0.60 | 2 | 0.45 | 0.36 | Liang et al., 2009 |
| EM78 | 0.75 | 3 | 0.26 | 0.32 | Liang et al., 2009 |
| EM87 | 0.56 | 4 | 0.03 | 0.44 | Liang et al., 2009 |
| GA133 | 0.53 | 3 | 0.08 | 0.38 | Hong et al., 2010 |
| GA156 | 0.58 | 4 | 0.04 | 0.39 | Hong et al., 2010 |
| GA21 | 0.51 | 6 | 0.34 | 0.58 | Hong et al., 2010 |
| GA24 | 0.56 | 3 | 0.03 | 0.52 | Hong et al., 2010 |
| GA26 | 0.68 | 4 | 0.08 | 0.43 | Hong et al., 2010 |
| GA28 | 0.82 | 3 | 0.07 | 0.26 | Hong et al., 2010 |
| GA8 | 0.70 | 2 | 0.01 | 0.33 | Hong et al., 2010 |
| TC1E5 | 0.73 | 3 | 0.25 | 0.36 | Moretzsohn et al., 2005 |
| TC7C6 | 0.47 | 5 | 0.08 | 0.61 | Moretzsohn et al., 2005 |
| TC5A6 | 0.87 | 5 | 0.01 | 0.23 | Moretzsohn et al., 2005 |
| GM1311 | 0.70 | 2 | 0.01 | 0.33 | Qin et al., 2012 |
| GM1445 | 0.53 | 2 | 0.11 | 0.37 | Qin et al., 2012 |
| GM1483 | 0.67 | 2 | 0.12 | 0.34 | Qin et al., 2012 |
| GM1577-1 | 0.57 | 4 | 0.09 | 0.50 | Nagy et al., 2009 |
| GM1577-2 | 0.59 | 2 | 0.06 | 0.37 | Nagy et al., 2009 |
| GM1713 | 0.50 | 3 | 0.01 | 0.48 | Nagy et al., 2009 |
| GM1742 | 0.53 | 2 | 0.01 | 0.37 | Qin et al., 2012 |
| GM1760 | 0.73 | 2 | 0.04 | 0.31 | Qin et al., 2012 |
| GM1798 | 0.59 | 2 | 0.11 | 0.37 | Qin et al., 2012 |
| GM1953 | 0.92 | 2 | 0.02 | 0.13 | Qin et al., 2012 |
| GM2032 | 0.49 | 4 | 0.33 | 0.48 | Qin et al., 2012 |
| GM2076 | 0.80 | 2 | 0.13 | 0.27 | Qin et al., 2012 |
| GM2084 | 0.62 | 3 | 0.16 | 0.37 | Nagy et al., 2009 |
| GM2106 | 0.45 | 3 | 0.42 | 0.56 | Qin et al., 2012 |
| GM2137 | 0.54 | 2 | 0.01 | 0.37 | Qin et al., 2012 |
| GM2156 | 0.90 | 3 | 0.05 | 0.18 | Qin et al., 2012 |
| GM2246 | 0.56 | 4 | 0.32 | 0.46 | Qin et al., 2012 |
| GM2259 | 0.70 | 2 | 0.21 | 0.33 | Qin et al., 2012 |
| GM2289 | 0.62 | 2 | 0.10 | 0.36 | Qin et al., 2012 |
| GM2337 | 0.59 | 2 | 0.01 | 0.37 | Qin et al., 2012 |
| GM2444-1 | 0.82 | 2 | 0.02 | 0.25 | Qin et al., 2012 |
| GM2444-2 | 0.99 | 2 | 0.01 | 0.01 | Qin et al., 2012 |
| GM2553 | 0.83 | 2 | 0.03 | 0.24 | Qin et al., 2012 |
| GM2557 | 0.83 | 3 | 0.05 | 0.25 | Nagy et al., 2009 |
| GM2571 | 0.54 | 2 | 0.06 | 0.37 | Nagy et al., 2009 |
| GM2589 | 0.52 | 3 | 0.01 | 0.46 | Qin et al., 2012 |
| GM2689 | 0.61 | 2 | 0.01 | 0.36 | Qin et al., 2012 |
| GM2690 | 0.56 | 2 | 0.24 | 0.37 | Qin et al., 2012 |
| GM2745 | 0.55 | 2 | 0.03 | 0.37 | Qin et al., 2012 |
| GM2788 | 0.55 | 3 | 0.1 | 0.38 | Qin et al., 2012 |
| GM654 | 0.69 | 2 | 0.51 | 0.34 | Qin et al., 2012 |
| GM678 | 0.57 | 2 | 0.02 | 0.37 | Qin et al., 2012 |
| GNB1062 | 0.84 | 2 | 0.30 | 0.23 | Wang et al., 2012 |
| GNB159 | 0.75 | 3 | 0.01 | 0.31 | Wang et al., 2012 |
| GNB320 | 0.67 | 2 | 0.01 | 0.35 | Wang et al., 2012 |
| GNB665 | 0.87 | 2 | 0.02 | 0.21 | Wang et al., 2012 |
| GNB716 | 0.68 | 3 | 0.02 | 0.37 | Wang et al., 2012 |
| GNB983 | 0.46 | 6 | 0.06 | 0.58 | Wang et al., 2012 |
| Seq16C7 | 0.8 | 3 | 0.01 | 0.31 | Ferguson et al., 2004 |
| HAS0055 | 0.85 | 2 | 0.01 | 0.22 | Zhang et al., 2012 |
| HAS0302 | 0.55 | 3 | 0.31 | 0.38 | Zhang et al., 2012 |
| HAS0313 | 0.55 | 2 | 0.19 | 0.37 | Zhang et al., 2012 |
| HAS0345 | 0.53 | 2 | 0.15 | 0.37 | Zhang et al., 2012 |
| HAS0428 | 0.59 | 3 | 0.31 | 0.38 | Zhang et al., 2012 |
| HAS0818 | 0.52 | 3 | 0.01 | 0.39 | Zhang et al., 2012 |
| HAS0869 | 0.83 | 2 | 0.06 | 0.24 | Zhang et al., 2012 |
| HAS0921 | 0.56 | 3 | 0.15 | 0.38 | Zhang et al., 2012 |
| HAS0969 | 0.62 | 3 | 0.15 | 0.38 | Zhang et al., 2012 |
| HAS1011 | 0.68 | 2 | 0.07 | 0.34 | Zhang et al., 2012 |
| IPAHM037 | 0.57 | 6 | 0.14 | 0.56 | Cuc et al., 2008 |
| IPAHM093 | 0.82 | 3 | 0.04 | 0.27 | Cuc et al., 2008 |
| IPAHM176 | 0.54 | 2 | 0.01 | 0.37 | Cuc et al., 2008 |
| IPAHM282 | 0.42 | 4 | 0.16 | 0.61 | Cuc et al., 2008 |
| IPAHM288 | 0.53 | 3 | 0.01 | 0.46 | Cuc et al., 2008 |
| IPAHM352 | 0.61 | 3 | 0.10 | 0.37 | Cuc et al., 2008 |
| IPAHM395 | 0.80 | 2 | 0.10 | 0.27 | Cuc et al., 2008 |
| IPAHM407A | 0.60 | 4 | 0.03 | 0.54 | Cuc et al., 2008 |
| IPAHM475 | 0.70 | 2 | 0.48 | 0.33 | Cuc et al., 2008 |
| IPAHM531 | 0.61 | 2 | 0.08 | 0.36 | Cuc et al., 2008 |
| IPAHM606 | 0.54 | 2 | 0.01 | 0.37 | Cuc et al., 2008 |
| PM204 | 0.82 | 3 | 0.02 | 0.28 | He et al., 2003 |
| PM419 | 0.56 | 2 | 0.26 | 0.37 | He et al., 2003 |
| PM42 | 0.90 | 2 | 0.03 | 0.16 | He et al., 2003 |
| PM54 | 0.57 | 3 | 0.01 | 0.39 | He et al., 2003 |
| PM675 | 0.65 | 5 | 0.09 | 0.43 | Yuan et al., 2010 |
| POCR349 | 0.83 | 2 | 0.10 | 0.24 | Tang et al., 2012 |
| POCR536 | 0.58 | 2 | 0.13 | 0.37 | Tang et al., 2012 |
| Seq14C11 | 0.34 | 9 | 0.05 | 0.67 | Ferguson et al., 2004 |
| Seq1B9 | 0.42 | 3 | 0.51 | 0.58 | Ferguson et al., 2004 |
| Seq2A5 | 0.39 | 5 | 0.06 | 0.59 | Ferguson et al., 2004 |
| Seq2E6 | 0.54 | 2 | 0.38 | 0.37 | Ferguson et al., 2004 |
| Seq2E8 | 0.84 | 3 | 0.06 | 0.25 | Ferguson et al., 2004 |
| Seq2F5 | 0.86 | 3 | 0.22 | 0.23 | Ferguson et al., 2004 |
| Seq2G4 | 0.45 | 4 | 0.03 | 0.55 | Ferguson et al., 2004 |
| Seq4E10 | 0.82 | 4 | 0.31 | 0.29 | Ferguson et al., 2004 |
| Seq4E8 | 0.57 | 2 | 0.12 | 0.37 | Moretzsohn et al., 2005 |
| Seq4G9 | 0.80 | 2 | 0.39 | 0.27 | Ferguson et al., 2004 |
| Seq5D1 | 0.52 | 3 | 0.04 | 0.5 | Ferguson et al., 2004 |
| Seq5D5 | 0.50 | 3 | 0.10 | 0.4 | Ferguson et al., 2004 |
| Seq7G2 | 0.60 | 3 | 0.10 | 0.49 | Ferguson et al., 2004 |
| TC11H6 | 0.64 | 4 | 0.03 | 0.47 | Moretzsohn et al., 2005 |
| TC19B11 | 0.69 | 3 | 0.20 | 0.37 | Wang et al., 2012 |
| TC19E1 | 0.56 | 7 | 0.08 | 0.59 | Wang et al., 2012 |
| TC23C8 | 0.56 | 3 | 0.39 | 0.51 | Wang et al., 2012 |
| TC2B9 | 0.88 | 2 | 0.11 | 0.19 | Moretzsohn et al., 2005 |
| TC2C7 | 0.87 | 2 | 0.01 | 0.20 | Moretzsohn et al., 2005 |
| TC2D6 | 0.74 | 4 | 0.05 | 0.32 | Moretzsohn et al., 2005 |
| TC2G5 | 0.35 | 7 | 0.35 | 0.75 | Moretzsohn et al., 2005 |
| TC31G11 | 0.67 | 2 | 0.03 | 0.34 | Wang et al., 2012 |
| TC3A12 | 0.77 | 3 | 0.10 | 0.29 | Moretzsohn et al., 2005 |
| TC3H2 | 0.57 | 4 | 0.09 | 0.52 | Moretzsohn et al., 2005 |
| TC4C11 | 0.88 | 2 | 0.01 | 0.19 | Moretzsohn et al., 2005 |
| TC4E10 | 0.64 | 3 | 0.01 | 0.45 | Moretzsohn et al., 2005 |
| TC4F10 | 0.83 | 4 | 0.05 | 0.28 | Moretzsohn et al., 2005 |
| TC4G10 | 0.59 | 3 | 0.32 | 0.40 | Moretzsohn et al., 2005 |
| TC4H2 | 0.78 | 2 | 0.02 | 0.29 | Moretzsohn et al., 2005 |
| TC6H3 | 0.54 | 2 | 0.01 | 0.37 | Moretzsohn et al., 2005 |
| TC7A2 | 0.50 | 3 | 0.22 | 0.50 | Moretzsohn et al., 2005 |
| Mean | 0.63 | 2.99 | 0.11 | 0.38 |  |
