# Supplementary material for: Genetic Diversity and Population Structure of the Major Peanut (Arachis hypogaea L.) Cultivars Grown in China by SSR Markers
Source: PLoS One. 2014 Feb 10;9(2):e88091. doi: 10.1371/journal.pone.0088091 (PMC3919752; doi:10.1371/journal.pone.0088091)
Supplement: Table S3 — Summary genetic statistics of peanut cultivars in different years. (DOC) [file pone.0088091.s005.doc]

**Table S3.** Summary genetic statistics of peanut cultivars in different years.

| **Years** | **NS** | **MAF** | **AN** | **GD** | **PIC** | **NRA(Ratio*)** |
| --- | --- | --- | --- | --- | --- | --- |
| Before 1970 | 18 | 0.63 | 2.5 | 0.14 | 0.38 | 14(77.8%) |
| 1971-1980 | 15 | 0.64 | 2.41 | 0.15 | 0.36 | 7(46.7%) |
| 1981-1990 | 33 | 0.66 | 2.64 | 0.12 | 0.37 | 16(48.5%) |
| 1991-2000 | 34 | 0.64 | 2.64 | 0.12 | 0.37 | 16(47.1%) |
| 2001-2010 | 96 | 0.65 | 2.83 | 0.14 | 0.37 | 42(43.8%) |

NS, Number of the samples; MAF, Major allele frequency; AN, Number of alleles per locus; GD, Gene diversity; PIC, Polymorphism information content; NRA, Number of rare alleles; Ratio*, Ratio of cultivars with rare allele.
