# Supplementary material for: Genetic Diversity and Population Structure of the Major Peanut (Arachis hypogaea L.) Cultivars Grown in China by SSR Markers
Source: PLoS One. 2014 Feb 10;9(2):e88091. doi: 10.1371/journal.pone.0088091 (PMC3919752; doi:10.1371/journal.pone.0088091)
Supplement: Table S4 — Pairwise estimates of Fst and Nei’s genetic distance based on 146 SSR loci among different years. (DOC) [file pone.0088091.s006.doc]

Table S4 Pairwise estimates of *F_st_* and Nei’s genetic distance based on 146 SSR loci among different years.

| Population | Before1970 | 1971-1980 | 1981-1990 | 1991-2000 | 2001-2010 |
| --- | --- | --- | --- | --- | --- |
| Before1970 |  | 0.05 | 0.07 | 0.06 | 0.04 |
| 1971-1980 | 0.18 |  | 0.02 | 0.02 | 0.03 |
| 1981-1990 | 0.21 | 0.13 |  | 0.01 | 0.03 |
| 1991-2000 | 0.20 | 0.11 | 0.10 |  | 0.00* |
| 2001-2010 | 0.20 | 0.12 | 0.12 | 0.08 |  |

Note: Genetic distance estimates appear above the diagonal and pairwise *F_st_* appears below the diagonal. 0.00* stands for the data being less than 0.005
